# Supplementary figures and images for: Pseudomonas aeruginosa displays a dormancy phenotype during long-term survival in water
Source: PLoS One. 2018 Sep 20;13(9):e0198384. doi: 10.1371/journal.pone.0198384 (PMC6147739; doi:10.1371/journal.pone.0198384)

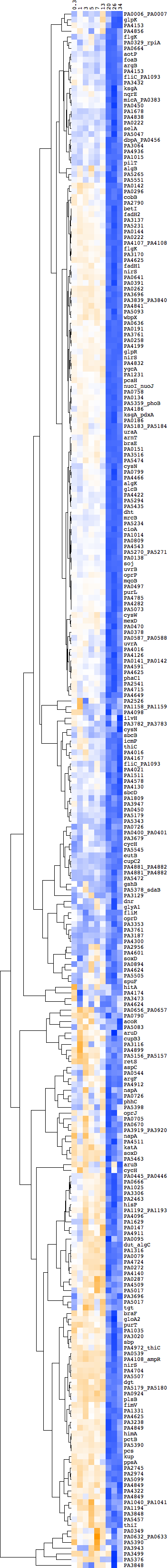

Supplement: S1 Fig — The PAO1 mini-Tn5-luxCDABE mutant library was inoculated into water and incubated at room temperature. At each time point the optical density (OD600) and luminescence (counts per second) was measured. Gene expression (CPS) readings were taken at day 0, 0.3, 1, 3, 5, 7, 13, 20, 26, and 34. Luminescence was divided by absorbance and fold changes were calculated based on the change in expression (CPS/OD600) compared to time 0. Cluster analysis was performed using Tree View and Cluster 3.0 software. Orange indicates repression, and blue indicates induced expression, relative to the time zero point. Genes with no change in expression are in white. This figure highlights genes expressed after one month in water. (PNG) [file pone.0198384.s001.png]

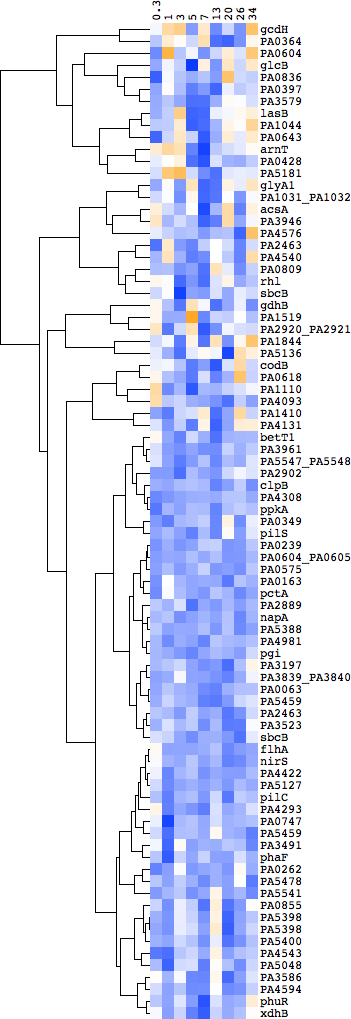

Supplement: S2 Fig — The PAO1 mini-Tn5-luxCDABE mutant library was inoculated into water and incubated at room temperature. At each time point the optical density (OD600) and luminescence (counts per second) was measured. Gene expression (CPS) readings were taken at day 0, 0.3, 1, 3, 5, 7, 13, 20, 26, and 34. Luminescence was divided by absorbance and fold changes were calculated based on the change in expression (CPS/OD600) compared to time 0. Cluster analysis was performed using Tree View and Cluster 3.0 software. Orange indicates repression, and blue indicates induced expression, relative to the time zero point. Genes with no change in expression are in white. This figure highlights genes expressed throughout the time course. (PNG) [file pone.0198384.s002.png]

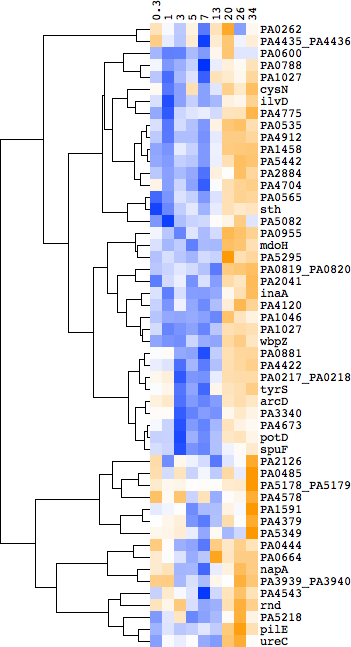

Supplement: S3 Fig — The PAO1 mini-Tn5-luxCDABE mutant library was inoculated into water and incubated at room temperature. At each time point the optical density (OD600) and luminescence (counts per second) was measured. Gene expression (CPS) readings were taken at day 0, 0.3, 1, 3, 5, 7, 13, 20, 26, and 34. Luminescence was divided by absorbance and fold changes were calculated based on the change in expression (CPS/OD600) compared to time 0. Cluster analysis was performed using Tree View and Cluster 3.0 software. Orange indicates repression, and blue indicates induced expression, relative to the time zero point. Genes with no change in expression are in white. This figure highlights genes expressed at early time points and then repressed at later time points. (PNG) [file pone.0198384.s003.png]

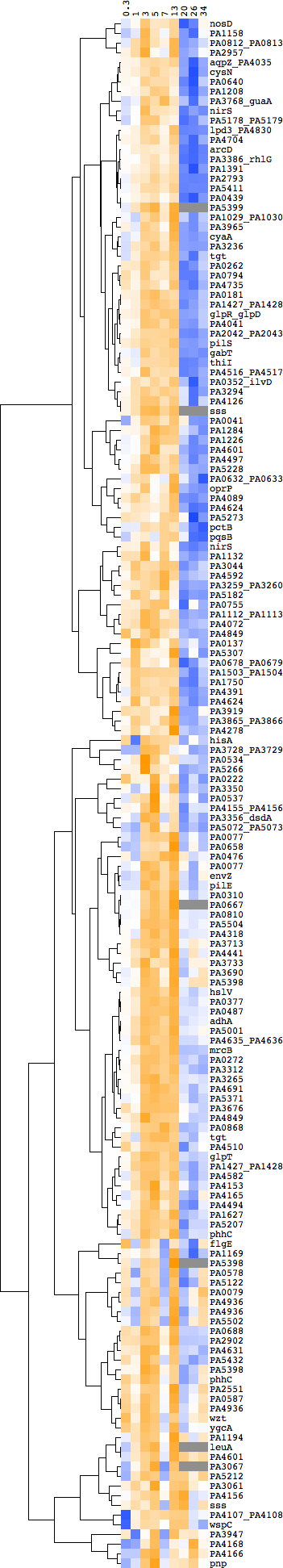

Supplement: S4 Fig — The PAO1 mini-Tn5-luxCDABE mutant library was inoculated into water and incubated at room temperature. At each time point the optical density (OD600) and luminescence (counts per second) was measured. Gene expression (CPS) readings were taken at day 0, 0.3, 1, 3, 5, 7, 13, 20, 26, and 34. Luminescence was divided by absorbance and fold changes were calculated based on the change in expression (CPS/OD600) compared to time 0. Cluster analysis was performed using Tree View and Cluster 3.0 software. Orange indicates repression, and blue indicates induced expression, relative to the time zero point. Genes with no change in expression are in white. This figure highlights genes expressed at later time points (close to one month). (PNG) [file pone.0198384.s004.png]
